# Dissection of Cancer Mutational Signatures with Individual Components of Cigarette Smoking

**DOI:** 10.1021/acs.chemrestox.3c00021

**Published:** 2023-03-28

**Authors:** Cécile Mingard, James N. D. Battey, Vakil Takhaveev, Katharina Blatter, Vera Hürlimann, Nicolas Sierro, Nikolai V. Ivanov, Shana J. Sturla

**Affiliations:** † Department of Health Sciences and Technology, ETH Zurich, Schmelzbergstrasse 9, Zürich, CH 8092, Switzerland; ‡ PMI R&D, Philip Morris Products SA, Quai Jeanrenaud 5, Neuchâtel, CH 2000, Switzerland

## Abstract

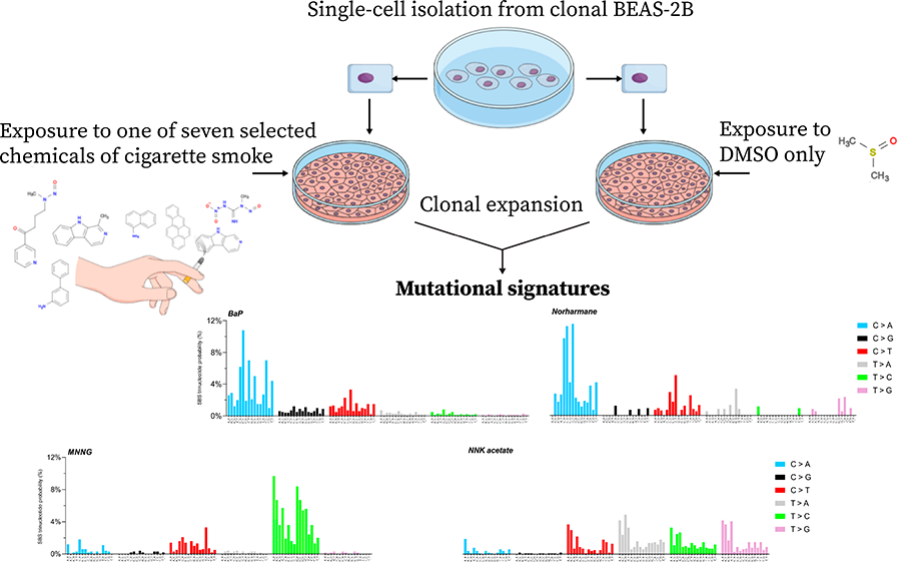

Tobacco smoke delivers a complex mixture of hazardous and potentially hazardous chemicals. Some of these may induce the formation of DNA mutations, which increases the risk of various cancers that display characteristic patterns of accumulated mutations arising from the causative exposures. Tracking the contributions of individual mutagens to mutational signatures present in human cancers can help understand cancer etiology and advance disease prevention strategies. To characterize the potential contributions of individual constituents of tobacco smoke to tobacco exposure-associated mutational signatures, we first assessed the toxic potential of 13 tobacco-relevant compounds by determining their impact on the viability of a human bronchial lung epithelial cell line (BEAS-2B). Experimentally derived high-resolution mutational profiles were characterized for the seven most potent compounds by sequencing the genomes of clonally expanded mutants that arose after exposure to the individual chemicals. Analogous to the classification of mutagenic processes on the basis of signatures from human cancers, we extracted mutational signatures from the mutant clones. We confirmed the formation of previously characterized benzo[*a*]pyrene mutational signatures. Furthermore, we discovered three novel mutational signatures. The mutational signatures arising from benzo[*a*]pyrene and norharmane were similar to human lung cancer signatures attributed to tobacco smoking. However, the signatures arising from *N*-methyl-*N*′-nitro-*N*-nitrosoguanidine and 4-(acetoxymethyl)nitrosamino]-1-(3-pyridyl)-1-butanone were not directly related to known tobacco-linked mutational signatures from human cancers. This new data set expands the scope of the *in vitro* mutational signature catalog and advances understanding of how environmental agents mutate DNA.

## 1 Introduction

Over a lifetime, humans are exposed to a complex array of chemicals. Some give rise to mutations as initiating events in cancer development. Cancer genomes therefore contain a record of environmental exposures and distinct mutational processes that shaped the tumor. In 2013, Alexandrov et al. applied a non-negative matrix factorization algorithm on millions of cancer mutations to reveal more than 20 unique mutational signatures.^1^ Now, the collection has been extended into a catalog of 60 single base substitutions (SBS), 11 doublet base substitutions (DBS), 18 small insertion/deletions (INDEL), and 31 copy number mutational signatures (https://cancer.sanger.ac.uk/signatures/).^2^ Each mutational signature is thought to correspond to a particular mutagenic process, and these discoveries have catalyzed efforts to elucidate the etiology of signatures and exploit their diagnostic potential for precision therapy and cancer prevention.

Two SBS (SBS 4 and 92), one DBS (DBS 2), and one INDEL (ID 4) signature were associated with tobacco smoking, and one SBS (Signature 29) was associated with tobacco chewing,^3,4^ on the basis of patient smoking history, known mutational mechanisms, and cancer tissue type. Nonetheless, the detailed attribution of the many mutagens in tobacco and their contributions to mutational signatures observed in cancers of tobacco users remains incomplete. Signature 4 is predominant in lung cancers and has a large proportion of C > A mutations. It has been attributed largely to benzo[*a*]pyrene (BaP), but the degree to which other chemicals in cigarette smoke might also contribute to mutational Signature 4 is not clear.

Exposing *in vitro* cell systems to defined compounds to extract mutational signatures in a hypothesis/validation loop model has proven to be a powerful tool for studying cancer etiologies.^5−10^ For example, analysis of mutations arising when human induced pluripotent stem cells were exposed to various environmentally relevant chemicals and then clonally expanded suggested that the *in vitro* mutational signatures from BaP, dibenzo[*a*,*h*]anthracene, 5-methylchrysene, and dibenz[*a*,*j*]acridine were similar to Signature 4.^5^ Indeed, cigarettes contain thousands of harmful chemicals, of which at least 60 are established carcinogens (e.g., polycyclic aromatic hydrocarbons [PAHs], *N*-nitrosamines, heterocyclic compounds, aromatic amines, and aldehydes).^11^ Understanding how these chemicals contribute to the tobacco smoking mutational landscape could help clarify how individual chemicals contribute to carcinogenesis and stimulate the development of strategies to reduce cancer risk.

The objective of this study was to characterize mutational signatures arising from individual constituents of tobacco smoke and evaluate how they relate to tobacco-associated cancer mutational signatures. We selected 13 chemicals in tobacco with structures associated with known classes of mutagens and first measured their cytotoxic effects on a human bronchial lung epithelial cell line (BEAS-2B) as a proxy to assess DNA-damaging capacity. Seven of these compounds decreased cell viability and were further characterized for their mutagenic potential with whole-genome sequencing. We compared the experimentally derived high-resolution mutational spectra with known SBS signatures. The results support the suitability of using BEAS-2B cells as a human lung-relevant model for mutagenesis and provide completely novel SBS signatures associated with tobacco constituents.

## 2 Materials and Methods

### 2.1 Chemicals

Harmane, norharmane, 1-naphthylamine, 3-aminobiphenyl, dibenzo[*a*,*h*]anthracene, benzo[a]anthracene, pyrene, benzo[*a*]pyrene, *N*-nitrosonicotine (NNN) (1 mg/mL in methanol), 4-(methylnitrosamino)-1-(3-pyridyl)-1-butanone (NNK) (1 mg/mL in methanol), and acrylonitrile (5 mg/mL in methanol) were purchased from Sigma (Burlington, MA) (SI Table 1). *N*-methyl-*N*′-nitro-*N*-nitrosoguanidine (MNNG) was purchased from TCI America (Portland, OR) (SI Table 1). 4-(Acetoxymethyl)nitrosamino]-1-(3-pyridyl)-1-butanone (NNK acetate) was purchased from Toronto Research Chemicals (North York, ON, Canada) (SI Table 1). Unless otherwise mentioned, all other chemicals were purchased from Sigma. All solid chemicals were dissolved in DMSO for stock concentrations.

### 2.2 Cell Culture and Clonal Parental Population Generation

The human bronchial epithelial cell line BEAS-2B (CRL-9609) was purchased from ATCC (Manassas, VA). Cells were tested regularly for mycoplasma contamination. BEAS-2B were cultured using a BEGM Bronchial Epithelial Cell Growth Medium BulletKit (Lonza, Basel, Switzerland) at 37 °C in a humidified atmosphere with 5% CO_2_. In order to obtain a clonal parental BEAS-2B cell line, cells were seeded at limited dilution (500 cells per 96-well plate). Cells were left to recover until they were sufficiently viable to transfer (4–8 days), after which wells were visually inspected to identify those containing a single cell. These clones were expanded and passaged to Primaria (Corning, Corning, NY) 6-well plates and then to Primaria 10 cm dishes. The clonal parental population was chosen on the criteria that the cells displayed the same morphology as BEAS-2B cells and had the same division time. DNA was extracted (see Section 2.6), and clonality of the population was confirmed by whole-genome sequencing. The resulting clonal parental BEAS-2B cell line was cultured in the same way as the regular BEAS-2B cell line.

### 2.3 Rat Liver Fraction S9Mix

Harmane, norharmane, 1-naphthylamine, 3-aminobiphenyl, benzo[*a*]pyrene, NNN, NNK, pyrene dibenzo[*a*,*h*]anthracene, acrylonitrile, and benzo[*a*]anthracene require metabolic activation by cytochrome P450 enzymes to give rise to reactive metabolites (SI Table 1). Therefore, cell exposure to the above-mentioned chemicals was done in the presence of an S9 mix. The S9 mix contained 33 mM KCl, 8 mM MgCl_2_, and 5 mM glucose-6-phopshate (Roche, Basel, Switzerland), as reported previously by Maron et al.,^12^ 2 mM NADP (Roche, prepared fresh), and 1% S9 from Aroclor 1254 treated rats (Trinova Biochem, Giessen, Germany).

### 2.4 Cell Viability Assay

Clonal BEAS-2B cells were seeded in Primaria 96-well plates (10,000 cells/well). The next day, the cells were exposed to increasing concentrations of chemicals or to the solvent control (0.1, 1% DMSO or 2% methanol) for 1 h (SI Table 1). Then, cells were washed with prewarmed PBS, and fresh growth medium was added. Cell viability was recorded 72 h after the start of the chemical exposure using the Cell-Titer Glo kit (Promega, Madison, WI), which measures intracellular ATP content and was performed according to the manufacturer’s instructions. GraphPad Prism software (GraphPad Inc., San Diego, CA) was used to model dose-inhibition curves and determine the dose that induced a 50% loss of intracellular ATP (half maximal inhibitory concentration, IC_50_).

### 2.5 Clonal Expansion Assay

Clonal parental BEAS-2B cells were seeded in Primaria 6-well plates (500,000 cells/well). The next day, cells were exposed for 1 h to each compound at a concentration equal to its IC_50_ value (S9 mix was also added for compounds requiring metabolic activation; for BaP a concentration below the apparent IC_50_ value was used to avoid concerns related to poor solubility) or to the respective DMSO vehicle control (SI Table 1). After exposure, the cells were left to recover for 5 days. Cells were then seeded at limited dilutions (100–500 cells/Primaria 96-well plate) to isolate single cells in the wells. Each well was carefully evaluated in the next days under a microscope, and wells were categorized having no cells, only one cell, or more than one cell. Wells containing a single cell were maintained with weekly media changes until the population had reached ∼20% confluency. The cells were then passaged to a Primaria 6-well plate and then finally to a Primaria 10 cm dish to extract DNA, after which the resulting cell pellet was frozen. For each compound and control, we expanded and sequenced five independent clones for whole-genome sequencing. To have independent clones, we ensured that the clones originated from different wells of the original 6-well plate used for cell exposure.

### 2.6 DNA Extraction and Illumina Sequencing

DNA was extracted from approximately 5 × 10^6^ cells per sample using a QIAamp DNA Mini kit (Qiagen, Hilden, Germany) according to the manufacturer’s protocol. DNA sequencing libraries were prepared with a Tecan Celero EZ DNA-Seq library preparation kit (Männedorf, Switzerland), starting from 100 ng of DNA, and with 20 min of enzymatic fragmentation and seven PCR amplification cycles. Libraries were quantified using NuQuant (Tecan) and normalized to 5 nM. The libraries were pooled and sequenced on Illumina NovaSeq6000 paired-end flow cells (Illumina, San Diego, CA) with 300 cycles using sequencing reagent kits.

### 2.7 Variant Calling and Mutational Spectra Analysis

Sequencing reads were filtered and trimmed to a maximum length of 150 base pairs using the software BBMap (version 38.79). The reads were subsequently aligned to the human genome (h38, Ensembl release 78) using the software “bwa” (version 0.7.17). Reads were filtered using Samtools (version 1.10), using the rule that reads must have none the following flags set: UNMAP, MUNMAP, SECONDARY, QCFAIL, and SUPPLEMENTARY. Read files were then deduplicated and downsampled to contain a comparable number of reads using GATK (version 4.1.6.0). Variant calling was performed with Freebayes (version 1.3.2), using options “–min-mapping-quality 30–min-base-quality 20–min-supporting-allele-qsum 0–genotype-variant-threshold 0”, whereby calling was performed on multiple samples grouped together as follows: group 1 – MNNG, NNK, and respective DMSO control; group 2 – harmane, norharmane, 1-naphthylamine, 3-aminobiphenyl, and respective DMSO control; group 3 – BaP and respective DMSO control. Within each group, colony-specific mutations were selected if they had been called exclusively for a given colony. Mutations were counted and analyzed using the R package MutationalPatterns (https://github.com/UMCUGenetics/MutationalPatterns).^13^ Mutation numbers including SBS, DBS, and INDELS in the respective control groups were subtracted from treatment mutation number values.

## 3 Results

### 3.1 Identifying Mutational Signatures in BEAS-2B Cells

Our goal was to identify novel mutational signatures induced by tobacco smoke compounds in a human-relevant model to clarify lung carcinogenesis mechanisms. We selected 11 chemicals found in cigarette smoke including PAHs (benzo[*a*]pyrene, benzo[*a*]anthracene, pyrene, and dibenzo[*a*,*h*]antracene), nitrosamines (NNN and NNK), heterocyclic amines (harmane and norharmane), aromatic amines (1-naphthylamine and 3-aminobiphenyl), and one acid derivative (acrylonitrile). We included two additional chemicals, namely, NNK acetate and MNNG, which model the two metabolic pathways for NNK activation, giving rise to pyridyloxobutylation or methylation of DNA, respectively. The selection criteria included evidence for mutagenicity that could contribute to mutation signatures found in tobacco-associated cancers. Simultaneously, the selected chemicals were either not characterized previously as a basis for mutational signatures or were considered nonmutagenic in the mutational signatures compendium of 79 environmental agents.^5^ Finally, benzo[*a*]pyrene and dibenzo[*a*,*h*]antracene were considered as positive controls. We used human lung bronchial BEAS-2B cells because they are noncancerous cells that can become malignant upon chemical exposure, which makes this cell line a relevant model to study aspects of lung carcinogenesis.^14^

In order to compare cellular responses to different compounds at concentration levels that induce similar cytotoxic responses, in line with previous *in vitro* mutational signature studies,^5,7^ we first determined an IC_50_ value for each chemical. Furthermore, since metabolic activation is required for genotoxicity of the majority of the compounds (SI Table 1) and BEAS-2B cells have low P450 expression,^15^ S9 mix from rat liver was used. Dibenzo[*a*,*h*]antracene, benzo[*a*]anthracene, pyrene, acrylonitrile, NNN, and NNK did not decrease cell viability at the tested concentrations (SI Figure 1); however, higher doses could not be tested due to poor solubility. Exposure of the cells to seven of the compounds (MNNG, NNK acetate, BaP, harmane, norharmane, 1-naphthylamine, and 3-aminobiphenyl) led to a systematic decrease in cell viability (SI Figure 2), and we decided to perform chemical exposure and clonal expansion studies with these compounds (Table 1).

**Table 1:**
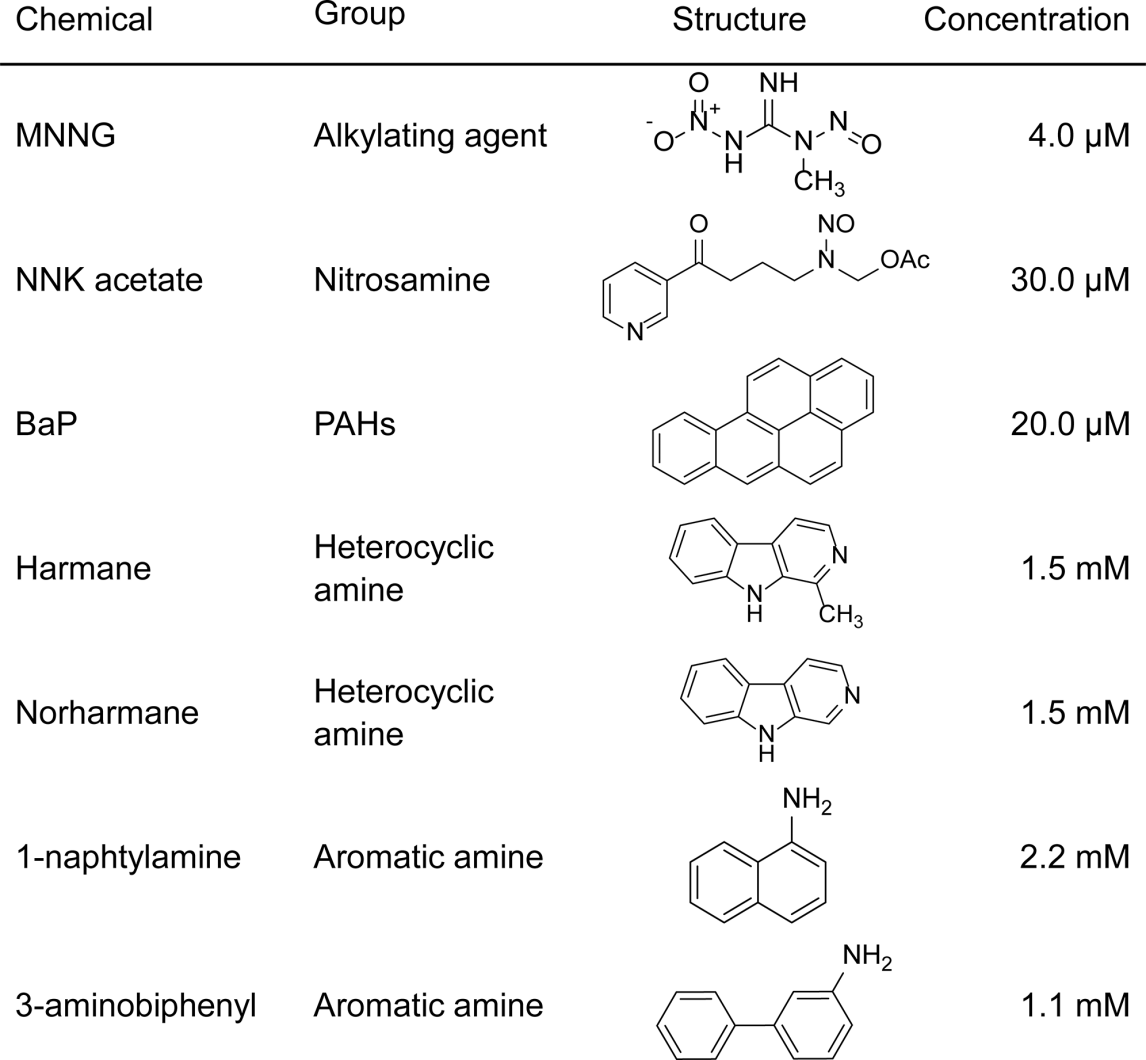
Chemicals Used in the Clonal Expansion Assay

To prevent false-positive identification of compound-induced mutations, we needed to account for variability in the unexposed BEA2-2B cells originating from normal bronchial epithelium of different noncancerous individuals. To minimize genetic variability, we first generated a clonal parental BEAS-2B cell population and assessed single nucleotide variants (SNVs) with whole genome sequencing. This SNV data set was used later to filter mutations not caused by the mutagen exposure. BEAS-2B proved to be cloneable, which is not the case for every cell type and was essential for this experiment.

**Figure 1:**
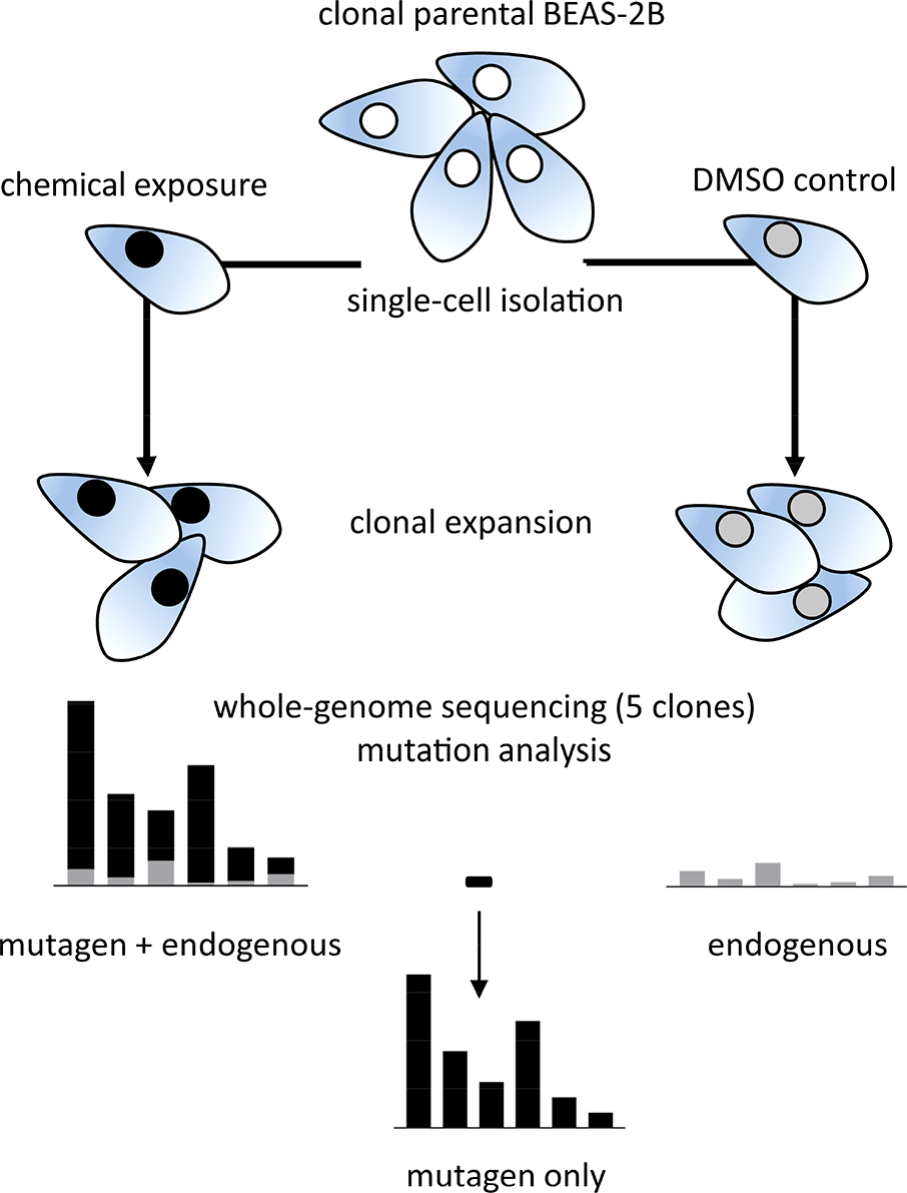
Experimental scheme of clonal expansion assay. BEAS-2B cells were either exposed to the compounds on the left or to their respective DMSO concentration control; then, single cells were isolated and clonally expanded for DNA extraction. Whole-genome sequencing was performed on five clones for each compound and controls. Mutations caused by the mutagens were extracted by subtracting endogenous control mutation values from compound exposure mutation values.

Parental clonal cells were exposed to chemicals at concentrations equal to their respective IC_50_ values for 1 h (Table 1 and Figure 1) and allowed to clonally expand. Five independent clones were raised and sequenced for each experimental condition, including respective DMSO vehicle controls. The mutations observed in the vehicle control groups are interpreted as endogenous mutations that arose during cell culture, likely due to oxidative DNA damage resulting from the high oxygen level in the atmosphere (∼21%) compared to physiological levels (∼3%),^16^ rather than DMSO, which has no known mutagenic activity at low concentrations.^17^ These endogenous mutations were subtracted from the mutagen group exposures to count SBS, DBS, and INDEL induced by each mutagen (Figure 1).

### 3.2 SBS Caused by Tobacco Smoke Mutagens

**Figure 2:**
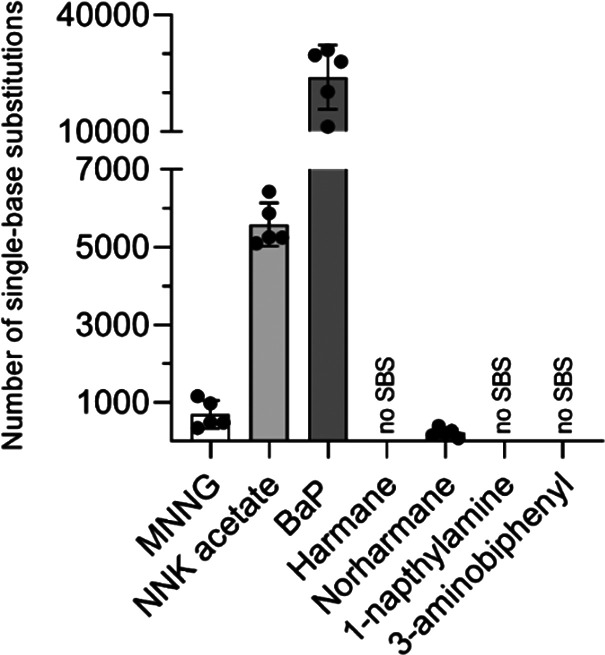
Number of single-base substitution for each tested compound after subtracting respective DMSO control number of single-base substitution number. Data represent the mean of five biological replicates ± SD.

We characterized SBS in the genomes of 45 clones arising from exposure to 7 compounds (Table 1) and 0.1 and 1% DMSO solvent control exposures. Both DMSO control clones had about 750 SBS in total, representing endogenous mutations emerging during clonal expansion. These were subtracted from the mutational spectra in chemically exposed clones (SI Figure 3). After subtraction, 4 out of the 7 tested compounds appeared to be mutagenic: norharmane (1.5 mM) induced on average 200 mutations, MNNG (4 μM) induced 700 mutations, NNK acetate (30 μM) induced 5500 mutations, and BaP (20 μM) induced 25,000 mutations (Figure 2). Conversely, harmane, 1-naphthylamine, and 3-aminobiphenyl were not found to generate SBS in BEAS-2B cells at the concentrations tested.

**Figure 3:**
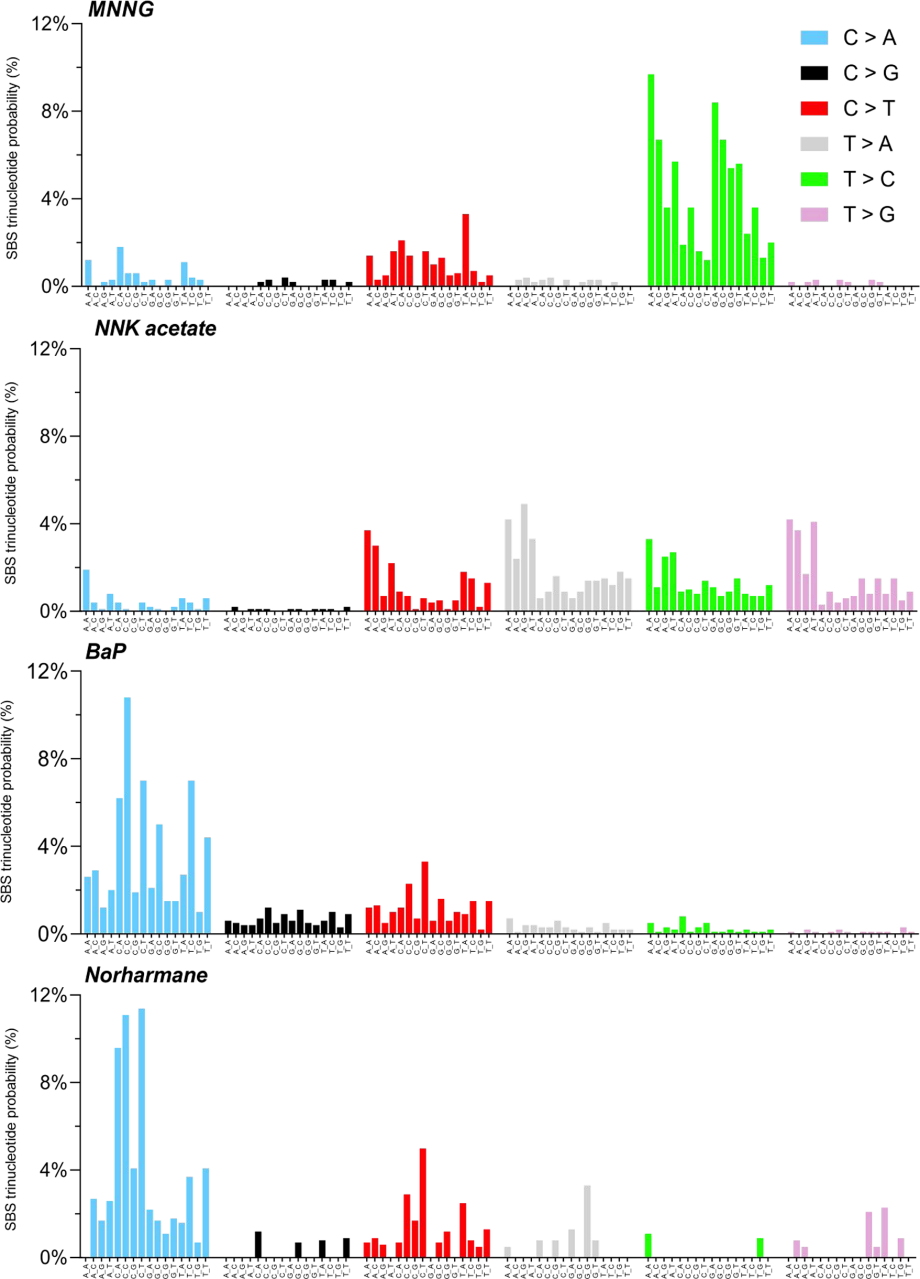
Single-base substitution probability in trinucleotide context for each mutagenic compound after subtracting respective DMSO control substitution number. Data represent the mean of five biological replicates.

SBS arising from exposure of BEAS-2B cells to MNNG, NNK acetate, BaP, and norharmane were classified by the 6 types of base substitutions (C > A, C > G, C > T, T > A, T > C, and T > G) in the 16 different flanking 5′- and 3′-base contexts. The relative frequency of each SBS in this classification was calculated to give rise to a 96 bar plot representing an experimentally derived high-resolution mutational spectrum (Figure 3).^1^ Each of the four compounds displayed a unique signature, suggesting distinct mechanisms of mutagenesis, which is consistent with the differences in the structures of the compounds (Table 1), their DNA damage products, and the impacts of cellular processes such as DNA damage repair and replicative bypass.

While there is strong precedent for the BaP-SBS4 relationship, the signatures arising from MNNG, NNK acetate, and norharmane are completely novel and—to our knowledge—have never been reported. The signature arising from MNNG exposure is characterized by a high proportion of T > C mutations preceded by a purine and to a lesser extent C > T (Figure 3). The signature arising from NNK acetate exposure has an equal representation of C > T, T > A, T > C, and T > G mutations, especially in contexts preceded by an A (Figure 3). The signature arising from norharmane is similar to the one arising from BaP, including a high prevalence of C > A and C > T mutations, but the trinucleotide profiles were not completely identical (Figure 3). Having extracted signatures for all four tobacco-related compounds, we have established a data set that can be compared to signatures extracted from human tumor genomes to understand cancer etiologies and for hazard characterization.

### 3.3 Comparison to Exposure or Cancer-Relevant Signatures

To date, only three COSMIC SBS signatures have been linked to tobacco exposure. Signature 4 was found in lung and head and neck cancers, while Signature 92 was found in bladder cancer and normal urothelium tissue from smokers (SI Figure 4).^2,18^ Signatures linked to tobacco chewing, such as Signature 29, was found in a variety of cancer tissue types in people who use chewing tobacco (SI Figure 4).^3^ The observation that Signature 4 was found only in lung, head, and neck cancer raised the question of why it could not be detected in any other cancer types attributed to tobacco smoking, such as liver cancer.^3^ It was postulated that the tobacco smoking mutation signature changes depending on the cell type due to differences in DNA damage formation, repair, and translesion synthesis (TLS) in tissues.

Characterizing further signatures that could be linked with tobacco constituents might help resolve unknown signature etiologies or be useful for assessing risks of different tobacco products or exposure scenarios. Thus, we compared our experimentally derived chemical-specific signatures to COSMIC cancer signatures^2^ and 41 environmental mutagen signatures^5^ using cosine similarity. This approach is used to evaluate the similarity between two signatures and gives a score ranging from 0 (no similarity) to 1 (identity), and while cut-offs in this value are arbitrary, we used 0.8 as a threshold for considering signatures as similar, consistent with previous studies.^19^

**Figure 4:**
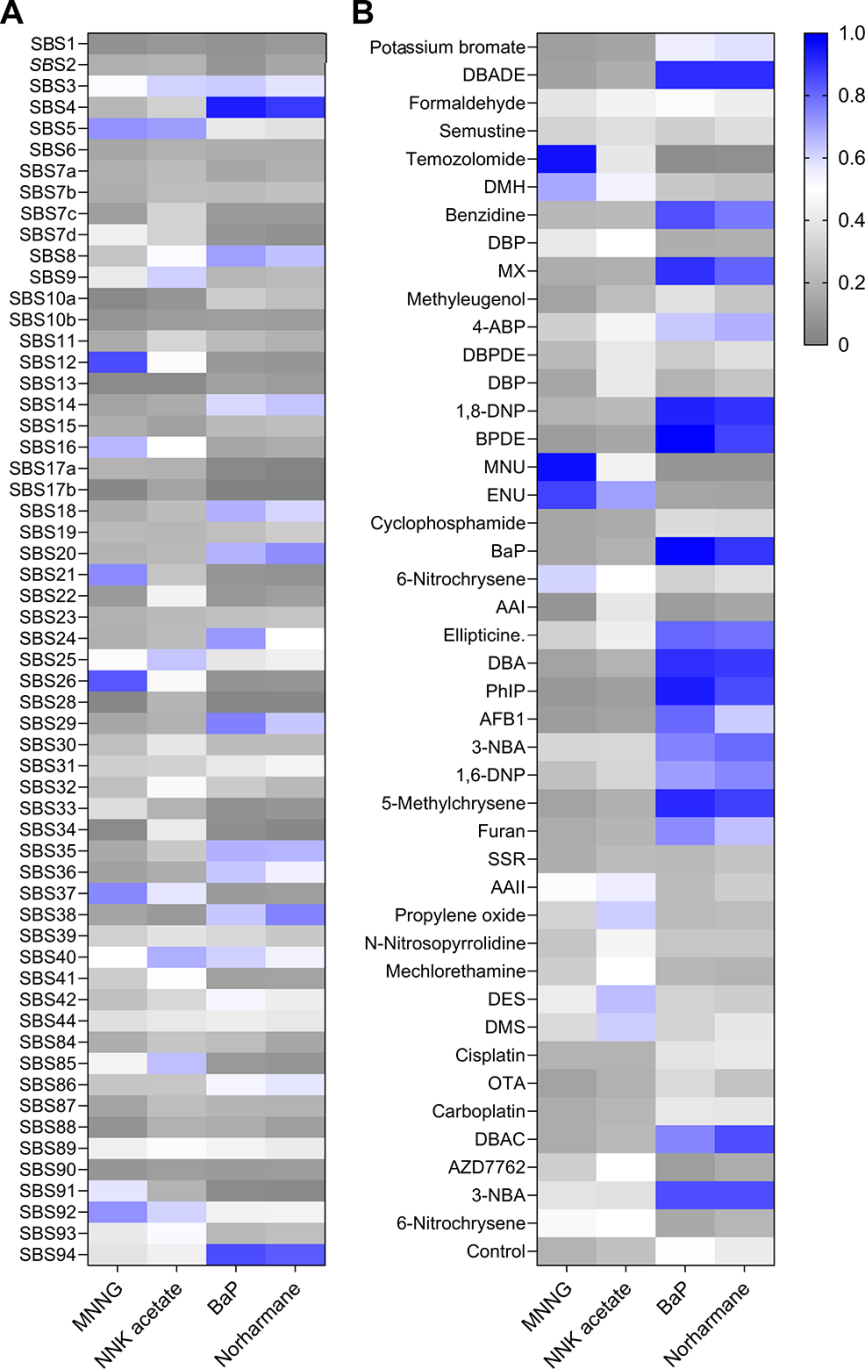
(A) Comparison using cosine similarity between MNNG, NNK acetate, BaP, and norharmane mutational signatures and COSMIC cancer mutational signatures. (B) Comparison using cosine similarity between MNNG, NNK acetate, BaP, and norharmane mutational signatures and published environmental mutagen signatures.

The MNNG signature uncovered in this study displayed high similarity with COSMIC Signatures 12 and 26 (0.83 and 0.81 cosine similarity, respectively) (Figure 4A). Both Signature 12 and 26 are characterized with high T > C mutation proportions (SI Figure 5), with the first signature having unknown etiology and the second linked to mismatch repair deficiency. Additionally, MNNG signature was >0.95 similar to a previously published alkylating agent signature of temozolomide and *N*-methyl-*N*-nitrosourea (MNU) and to a lesser extent (0.85) with the *N*-ethyl-*N*-nitrosourea (ENU) signature (Figure 4B).^5^ However, it was surprising that the majority of mutations were T > C and not C > T due to expected mispairing *O*^6^-meG with thymine, which could be explained by a rather high activity of the repair enzyme *O*^6^-methylguanine-methyl transferase (MGMT) in BEAS-2B cells as well as in human induced pluripotent stem cells (hiPSCs) used in the environmental mutagen signature study.^5^ The reason the potent mutagen MNNG did not yield any signature in the hiPSC study is unclear.^5^

BaP and norharmane gave rise to similar signatures, and the cosine comparisons were also similar. They both had high cosine similarity with Signature 4 (0.93 and 0.87, respectively) and with Signature 94 (0.84 and 0.81, respectively). The signature arising from NNK acetate was not similar to any of the COSMIC signatures or environmental mutagen signatures (Figure 4A,B).

### 3.4 BaP-Typical INDEL and DBS Signatures

We further looked at DBS and INDEL signatures as they could reveal other mutagenic mechanisms induced by environmental mutagens. DBS are the results of two bases mutated next to each other; the likelihood of this event occurring randomly is low,^20^ but increased DBSs have been associated with certain enzymes’ activities and exposure to genotoxic compounds.^2^ In the case of DNA-damaging agents, it is not clear whether the presence of DNA damage alters the likelihood of the adjacent base to be damaged or whether TLS polymerase’s low fidelity introduces patches of errors during replication. INDEL are the gain or loss of rather small DNA fragments ranging from 1–50 bp; they can occur because of strand slippage during replication^21^ or nonhomologous end-joining repair following double-strand breaks.^22^ INDEL are likely to occur in consecutive identical DNA base sequences called homopolymers.

**Figure 5:**
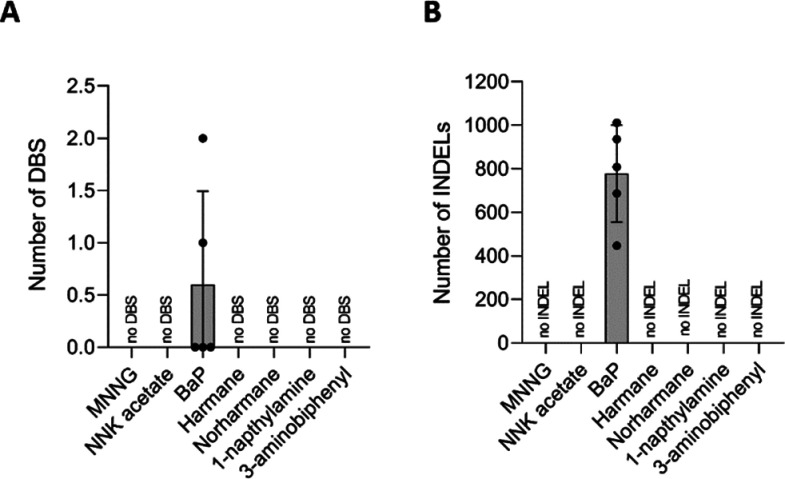
(A) Numbers of DBS for each tested compound after subtracting respective DMSO control DBS numbers. (B) Number of INDEL for each tested compound after subtracting respective DMSO control INDEL numbers. Data represent the mean of five biological replicates ± SD.

BaP was the only compound that led to increased numbers of DBS and INDEL. DBS were absent in three of BaP clones, and only one or two DBS were found in the remaining two BaP clones (Figure 5A). These DBS were characterized by CC > AA and CC > TA types (SI Figure 6), which were previously reported in the already published DBS tobacco smoking signature (SI Figure 7). Furthermore, there was a high number of INDEL induced by BaP in all samples (Figure 5B). BaP insertions and deletions were classified based on cancer INDEL mutational signature (SI Figures 6 and 7) and characterized by 1bp C and T deletions and 1bp T insertions, which resembles ID3 signature attributed to tobacco smoking (SI Figure 7).

## 4 Discussion

In this study, we dissected individual high-resolution mutational profiles of components of tobacco smoke, using a human lung cell line. We established that BEAS-2B cells, a human lung epithelial cell line with phenotypically normal characteristics, can be cloned and sequenced after chemical exposure as a model for the study of potential lung mutagens. We observed the formation of known benzo[*a*]pyrene (BaP) signatures (SBS, DBS, and INDEL), confirmed here for the first time in a human lung cell line. Moreover, we detected three novel SBS signatures caused by the nitrosamine metabolite 4-(acetoxymethyl)nitrosamino]-1-(3-pyridyl)-1-butanone (NNK acetate), the alkylating agent *N*-methyl-*N*′-nitro-*N*-nitrosoguanidine (MNNG), and the heterocyclic amine norharmane (Table 2). Comparing these signatures to the ones extracted from human tumor genomes could explain the mutational landscape in smoking-related cancers, suggesting etiologies for cancer Signatures 94 and 12 so far considered of unknown origin.

**Table 2:**
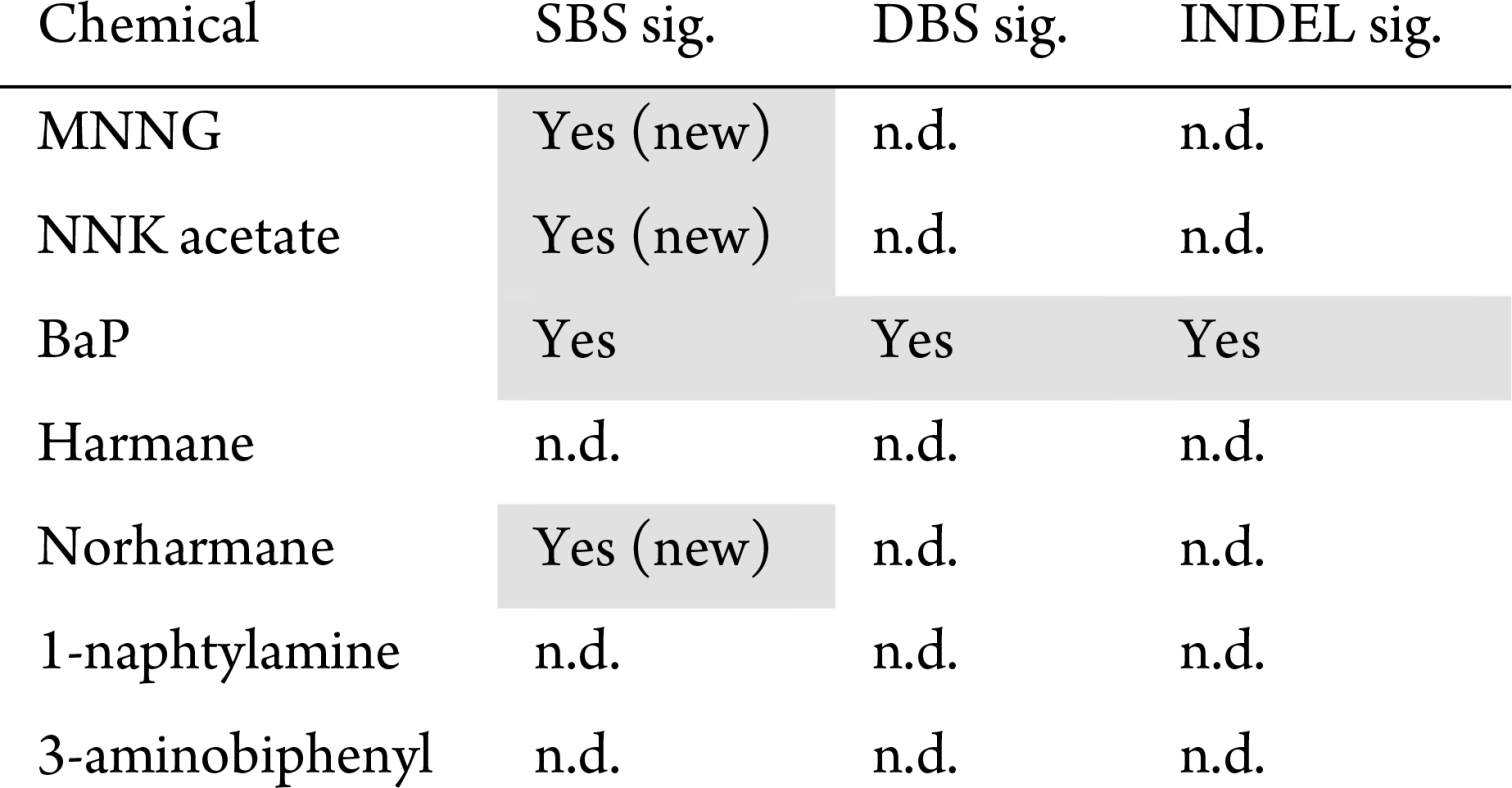
Summary of Mutational Signatures for Tested Compounds^a^

a n.d. = not detected.

Extracting mutational signatures from human cells exposed *in vitro* to defined chemicals has emerged as a powerful strategy to study cancer etiologies.^5−10^ There is a need to expand *in vitro* models for mutational signature studies in order to understand differences arising from varied physiological characteristics and as a basis for research in diverse areas of cancer prevention and therapy.^5,7,8,23,24^ As an example, the MNNG mutational signature experimentally observed here in BEAS-2B cells was characterized by T > C mutations most likely caused by *O*^4^-meT.^25^ Similar transition mutations were also observed for temozolomide, MNU, and ENU in hiPSCs.^5^ However, in mouse models, MNNG and MNU caused largely C > T mutations, consistent with *O*^6^-meG as a dominant basis of mutation.^7,10^ These variations may be associated with interspecies or intertissue differences in repairing particular DNA adducts.

The large number of mutations observed for cells exposed to BaP (around 25,000 SBS, up to 2 DBS and around 800 INDEL) is in line with its well-established mutagenicity.^5,10,26^ Further, its SBS signature, characterized mostly by C > A followed by C > T mutations (Figure 3), is consistent with DNA alkylation by the BaP metabolite BaP-7,8-dihydrodiol-9,10-epoxide (BPDE), forming *N*^2^-BPDE-dG,^27,28^ and its recently reported DNA damage signature.^52^ We also observed that norharmane, another PAH, induced a similar signature, with a high prevalence of C > A and C > T mutations (Figure 3), which expands the so-far limited evidence of the chemical’s mutagenicity. In previous reports, norharmane appeared to promote aniline-associated carcinogenesis, for instance, by forming the dG-*C*^*8*^-aminophenylnorharmane adduct^29−34^ and was considered a weak mutagen by itself in certain bacterial strains only.^35^ Although its mutational signature was highly similar to the one induced by BaP, this contributes to a relatively smaller proportion of tobacco-smoke-induced mutations (Figure 2). Nevertheless, the results support that mutagenic processes from related chemicals ultimately can result in similar signatures and may suggest that norharmane forms as yet uncharacterized adducts similar to BaP.

The novel signatures arising from MNNG and NNK acetate were extremely different from those arising from norharmane and BaP with regards to the frequencies of mutations in trinucleotide contexts. For MNNG, there was a high proportion of T > C mutations preceded by a purine and to a lesser extent C > T (Figure 3). Although MNNG can methylate various nucleobase positions,^36^ the mutational signature likely is due to the high mutagenic potential of *O*^6^-methylguanine (*O*^6^-meG) and *O*^4^-methylthymine (*O*^4^-meT).^25,37^ For NNK acetate, the new signature had equal proportions of C > T, T > A, T > C, and T > G mutations (Figure 3) in line with pyridyloxobutylation of guanine and thymine by NNK, giving rise to *O*^6^-pyridyloxobutylguanine (*O*^6^-pobG) and *O*^2^-pyridyloxobutylthymine (*O*^2^-pobT).^38−41^*O*^6^-pobG was shown to produce mainly CG > TA mutations in human cells,^42^ whereas *O*^2^-pobT induced TA > AT and to a smaller extend TA > CG and TA > GC.^43^ Relating these mutational signatures to those extracted from human tumors could help resolve molecular origins of cancer caused by smoking.

An important limitation concerns the concentrations of chemicals used in this study, which are substantially higher than real cigarette smoking-related exposures (e.g BaP, 8.5–17.6 ng/cigarette; NNK, 110–133 ng/cigarette; norharmane, 5.7 μg/cigarette).^11,35^ BaP, MNNG, and NNK acetate were used at micromolar levels, and for norharmane, the concentration was even as high as 1.5 mM. One factor contributing the high concentration was the standardization to equally cytotoxic concentrations. Nonetheless, cancer genomes evolve over long-term repeated exposure to significantly lower exposure levels. In these experiments, we effectively generated mutations from a single exposure, which is conversely rather high. Similarly, it has been shown also *in vivo* for aflatoxin that mutational signatures can arise from even a single exposure, again at a relatively high concentration (6 mg/kg).^24^ As yet, concentration–response and temporal (i.e., acute vs repeated exposure) relationships in the context of mutational signatures are not well-understood.

While comparing the experimentally observed signatures to the ones extracted from human tumor genomes, it was not surprising that the BaP signature strongly resembles Signature 4 (Figure 4A), which has been attributed to tobacco smoking.^3,4^ Moreover, this comparison suggested a possible basis explaining the currently unknown etiology of Signature 94, recently found in colorectal cancer with a highly sensitive signature extractor tool.^4^ Specifically, the high similarity of Signature 94 to the BaP and norharmane signatures (Figure 4A) indicates tobacco smoking etiology, which is in line with the established causal relationship between smoking and colorectal cancer.^44^ As another example of explaining cancer etiology by the generated data, Signatures 12 and 26 might arise from DNA alkylating agents forming *O*^4^-meT damage due to the fact that these cancer signatures resemble the MNNG-induced signature with dominating T > C transition, and Signature 26 has already been linked to the deficiency of mismatch repair that can recognize mispaired *O*^4^-meT.^45^ As for the NNK-acetate-induced signature, it is striking that the activated form of NNK, which is considered a strong tobacco-specific carcinogen,^46−48^ did not give rise to genomes with SBS signatures associated with human cancer or environmental exposures (Figure 4A,B) but instead an as yet unknown mutational profile. The findings presented here for tobacco carcinogens suggest how exposure to diverse chemical structures may shape the landscape of mutations caused by tobacco smoking.

Determining experimental mutational signatures is a useful strategy to understand how certain chemicals of tobacco smoke contribute to cancers. This approach may be also valuable to evaluate how alterations in constituents of smoking-related exposure could be introduced to reduce cancer risk. An additional translational relevance could involve biomonitoring for corresponding mutational signatures *in vivo* for early cancer diagnostics.^49^ Nonetheless, cigarette smoke contains a mixture of thousands of compounds,^11^ whereas in this study, we investigated seven chemicals and successfully identified signatures for four of them (Table 1). Thus, significant further work and additional data are needed in this area, focusing on large numbers of individual chemicals, their mixtures, and physiologically relevant cell models and exposure schemes (i.e., single high concentration exposure vs repeated lower concentrations) to dissect the contributors of complex exposures to the etiology of human cancers, and potentially linking these with genome-wide maps of DNA adducts or DNA damage signatures.^50−52^

## Data Availability

Sequencing data is deposited at the European Nucleotide Archive (https://www.ebi.ac.uk/ena/browser/home), project number PRJEB60585.

## Supporting Information Available

VCF files (vcf.gz files and Sample_VCF_codes.xlsx) (ZIP)
Figures of cell viability assays, SBS numbers for each exposure condition, COSMIC SBS mutation signatures, DBS and INDEL numbers for BaP, and COSMIC DBS and INDEL signatures and table of reagent sources and experimental exposure summary (PDF)

## Abbreviations Used

BaP: benzo[*a*]pyrene
BEAS-2B: human bronchial epithelial cells
BPDE: BaP-7,8-dihydrodiol-9,10-epoxide
ENU: *N*-ethyl-*N*-nitrosourea
hiPSC: human induced human pluripotent stem cell
MGMT: *O*^6^-methylguanine-methyl transferase
MNNG: *N*-methyl-*N*′-nitro-*N*-nitrosoguanidine
MNU: *N*-methyl-*N*-nitrosourea
NNK: 4-(methylnitrosamino)-1-(3-pyridyl)-1-butanone
NNK acetate: 4-(acetoxymethyl)nitrosamino]-1-(3-pyridyl)-1-butanone
NNN: *N*-nitrosonicotine
*O*^6^-meG: *O*^6^-methylguanine
*O*^4^-meT: *O*^4^-methylthymine
*O*^6^-pobG: *O*^6^-pyridyloxobutylguanine
*O*^2^-pobT: *O*^2^-pyridyloxobutylthymine
SNV: single nucleotide variant
TLS: translesion synthesis
