# Supplementary material for: Dissection of Cancer Mutational Signatures with Individual Components of Cigarette Smoking
Source: Chem Res Toxicol. 2023 Mar 28;36(4):714–23. doi: 10.1021/acs.chemrestox.3c00021 (PMC10114081; doi:10.1021/acs.chemrestox.3c00021)
Supplement: Supplementary file 2 — tx3c00021_si_002.pdf [file tx3c00021_si_002.pdf]

Supporting Information for

Dissection of cancer mutational signatures with individual components of cigarette smoking

Cécile Mingard[1], James N.D. Battey[2], Vakil Takhaveev[1], Katharina Blatter[1], Vera Hürlimann[1], Nicolas Sierro[2], Nikolai V. Ivanov[2], Shana J. Sturla[1*]

[1]ETH Zurich, Department of Health Sciences and Technology, Schmelzbergstrasse 9, Zürich, CH 8092

[2]PMI R&D, Philip Morris Products SA, Quai Jeanrenaud 5, Neuchâtel, CH 2000

*Corresponding author: Shana J. Sturla; Schmelzbergstrasse 9, 8092 Zürich, Switzerland; Tel.: +41 44 632 91 75; Email: sturlas@ethz.ch

Table of contents:

- SI Figures 1-7, pages S2-S5
- SI Table 1, page S6

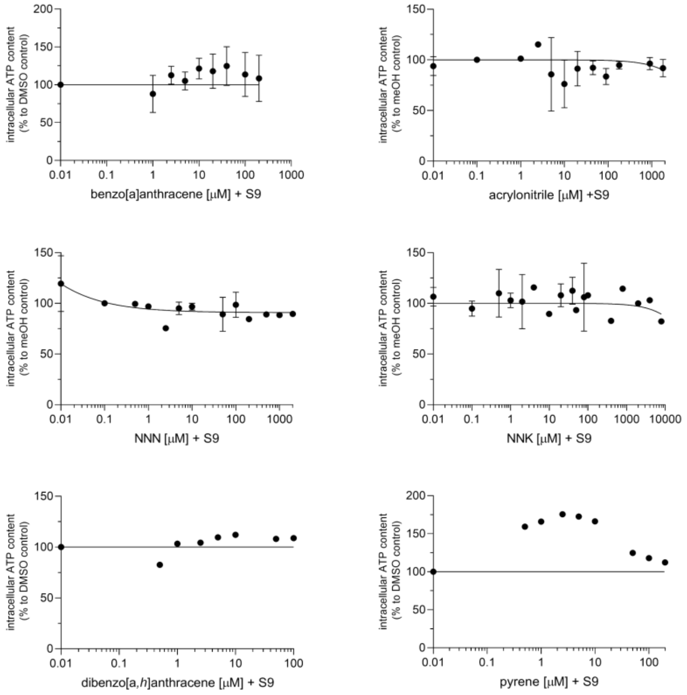

SI Figure 1: Cell viability assessment by measuring intracellular ATP levels. BEAS-2B cells were exposed to each compound for 1 hour and left to recover for 5 days. Data represent the mean normalized to DMSO or methanol controls of at least one biological replicate. Error bars indicate +/- SD.

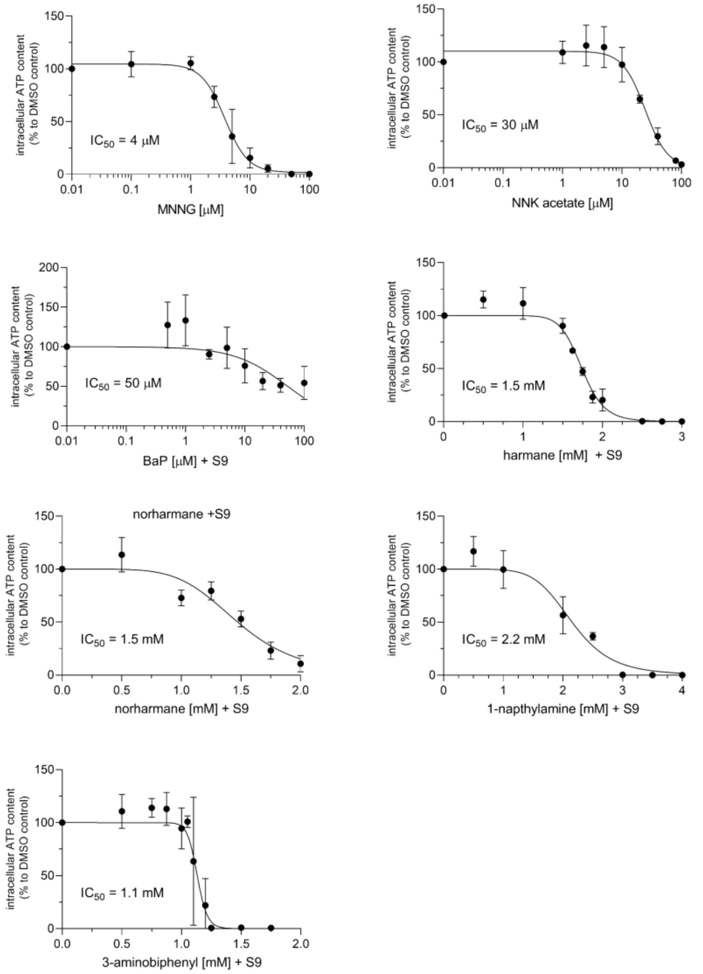

SI Figure 2: Cell viability by measuring intracellular ATP levels. BEAS-2B cells were exposed to each compound for 1 hour and left to recover for 5 days. Data represent the mean normalized to DMSO controls of at least three biological replicate. Error bars indicate +/- SD.

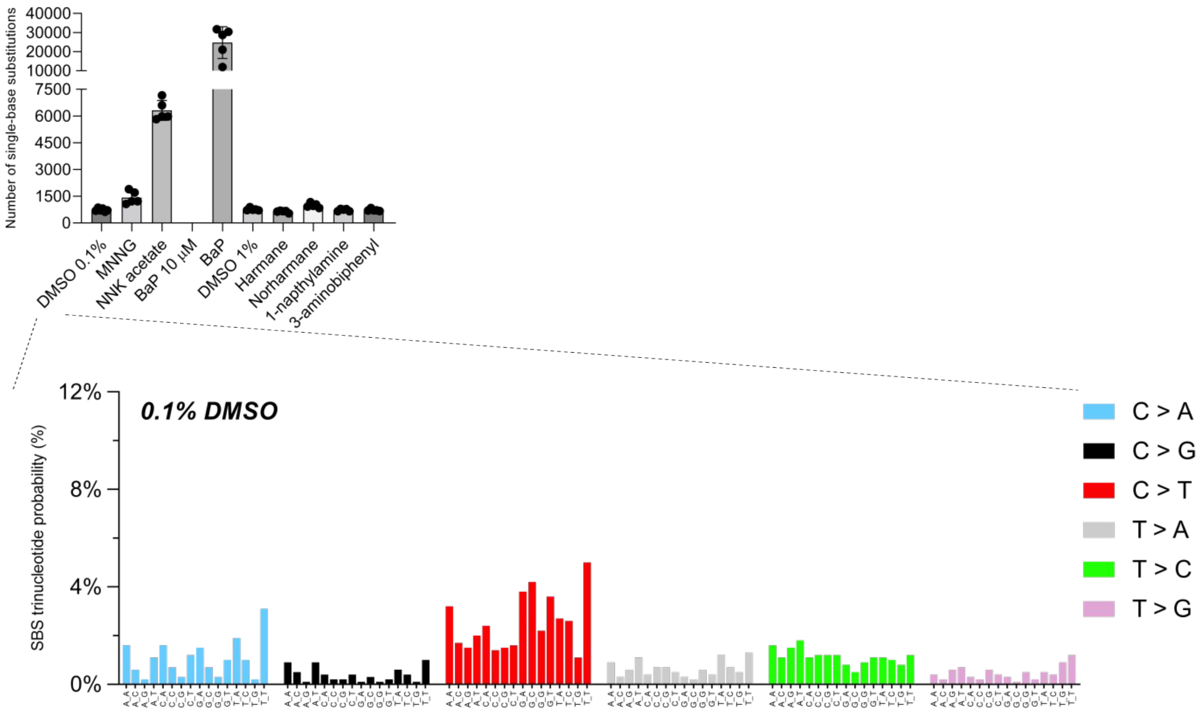

SI Figure 3: Single-base substitution number for each exposure conditions. Single-base substitution probability in trinucleotide context for 0.1% DMSO control. Data represent the mean of five biological replicates.

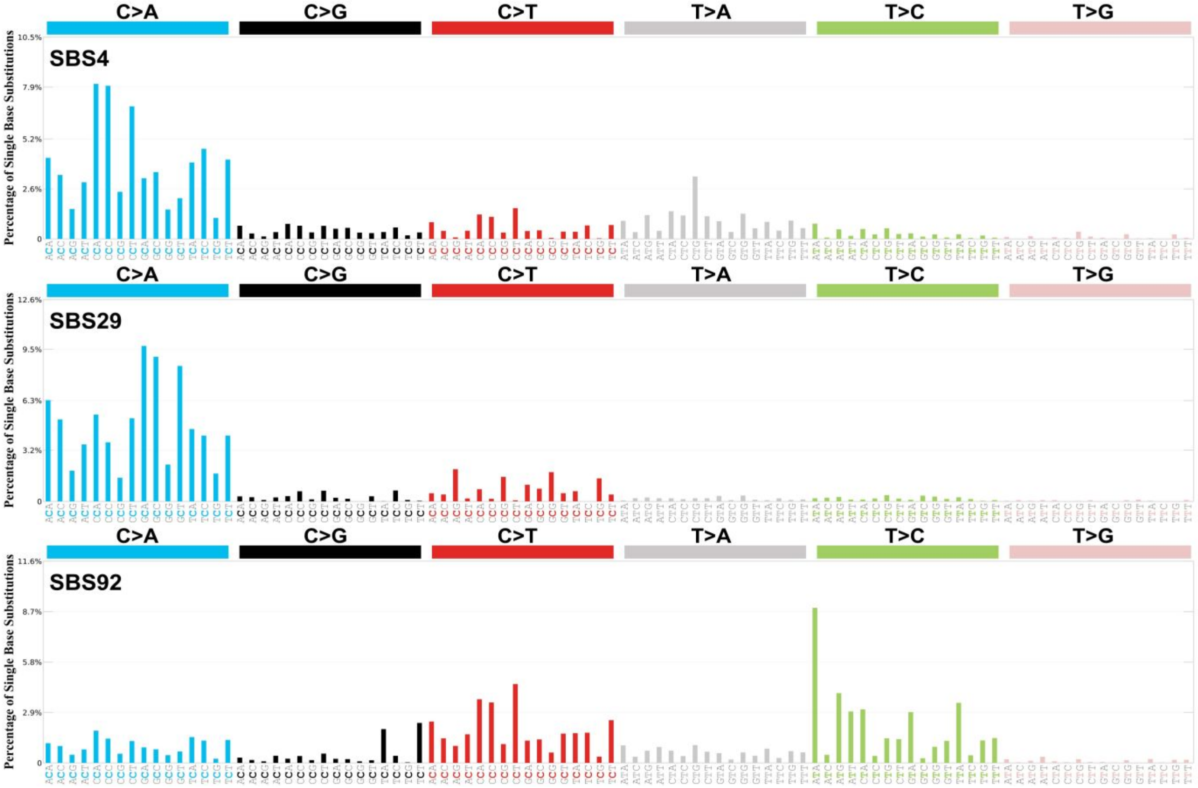

SI Figure 4: COSMIC SBS mutational Signature 4, 29 and 92 downloaded from: https://cancer.sanger.ac.uk/signatures/

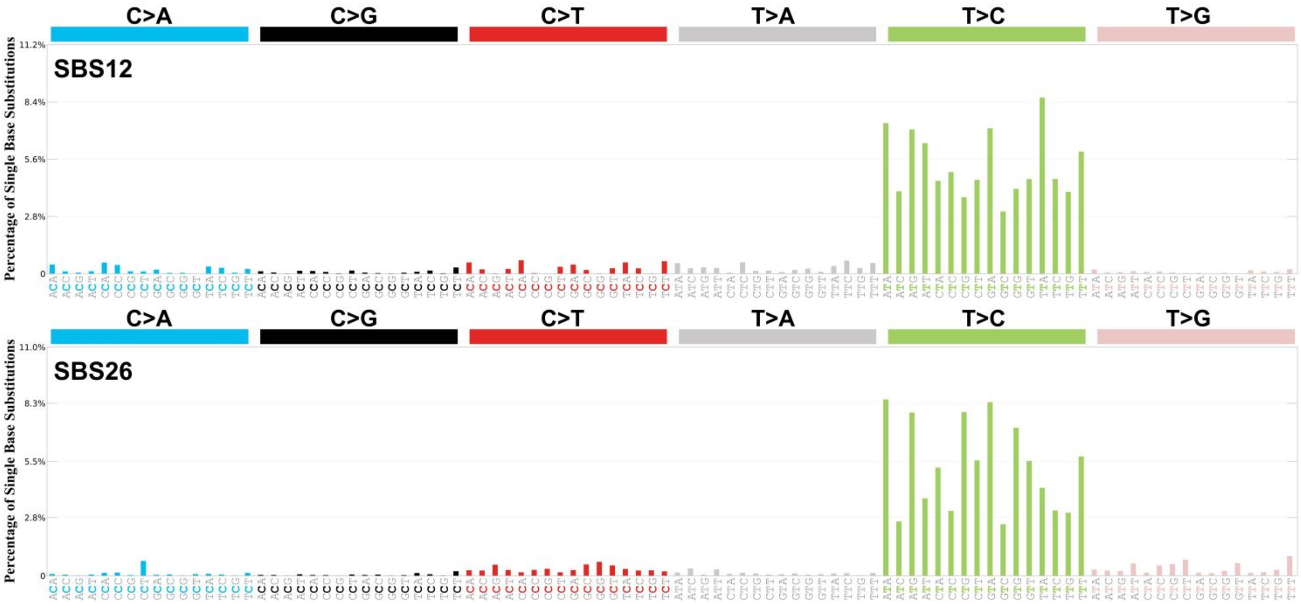

SI Figure 5: COSMIC SBS mutational Signature 12 and 26 downloaded from: https://cancer.sanger.ac.uk/signatures/

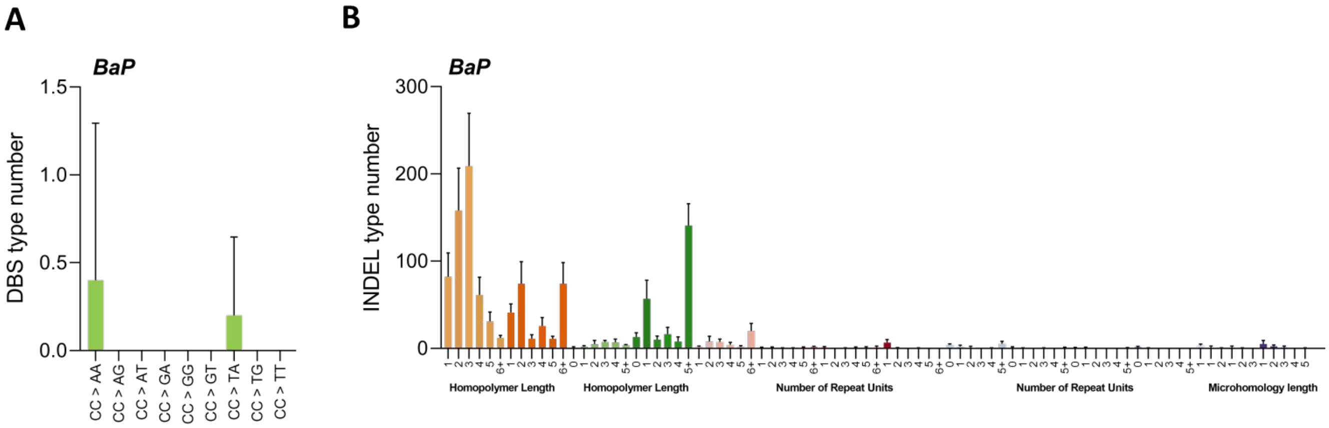

SI Figure 6: (A) Double-base substitution number for BaP after subtracting respective DMSO control double-base substitution number. (B) INDEL number for BaP after subtracting respective DMSO control INDEL number. Data represent the mean of five biological replicates +/- SD.

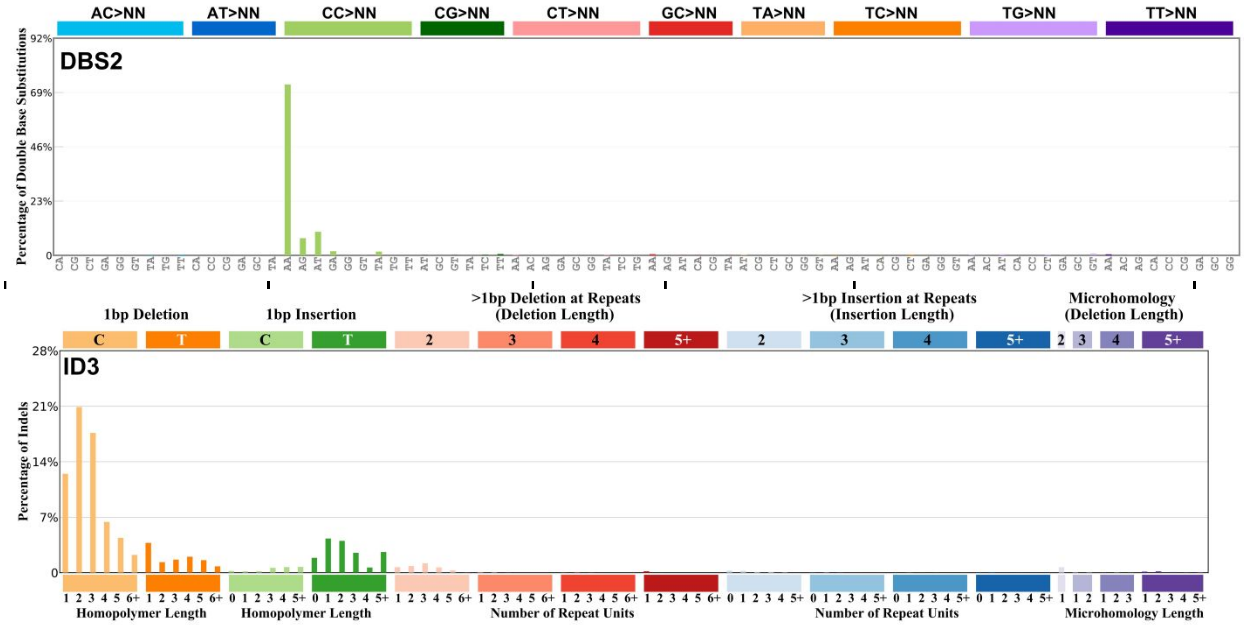

SI Figure 7: COSMIC mutational Signature DBS2 and ID3 downloaded from: https://cancer.sanger.ac.uk/signatures/

1-napthylamine	Sigma (N9005-25G)	Yes	1% DMSO	Yes
3-aminobiphenyl	Sigma (716448-1G)	Yes	1% DMSO	Yes
Acrylonitrile	Sigma (40003)	Yes	2% methanol	No (absence of cytotoxicity)
BaP	Sigma (B1760-100MG)	Yes	0.1% DMSO	Yes
Benzo[a]antracene	Sigma (B2209-1G)	Yes	0.1% DMSO	No (absence of cytotoxicity)
Dibenzo[a,h]antracene	Sigma (48574)	Yes	0.1% DMSO	No (absence of cytotoxicity)
Harmane	Sigma (103276-1G)	Yes	1% DMSO	Yes
MNNG	TCI chemical (M0527)	No	0.1% DMSO	Yes
NNK acetate	Toronto Research chemical (A167550)	No	0.1% DMSO	Yes
NNK	Sigma (N-076-1ML)	Yes	2% methanol	No (absence of cytotoxicity)
NNN	Sigma (N-075-1ML)	Yes	2% methanol	No (absence of cytotoxicity)
Norharmane	Sigma (N6252-100MG)	Yes	1% DMSO	Yes
Pyrene	Sigma (185515-1G)	Yes	0.1% DMSO	No (absence of cytotoxicity)

SI Table 1: Reagent sources and experimental exposure summary
